# Global link between deformation and volcanic eruption quantified by satellite imagery

**DOI:** 10.1038/ncomms4471

**Published:** 2014-04-03

**Authors:** J. Biggs, S. K. Ebmeier, W. P. Aspinall, Z. Lu, M. E. Pritchard, R. S. J. Sparks, T. A. Mather

**Affiliations:** 1 School of Earth Sciences, University of Bristol, Queen’s Road, Bristol BS8 1RJ, UK; 2 Roy M. Huffington Department of Earth Sciences, Southern Methodist University, P.O. Box 750395, Dallas, Texas 75275-0395, USA; 3 Department of Earth and Atmospheric Sciences, Cornell University, Ithaca, New York 14853, USA; 4 Department of Earth Sciences, University of Oxford, South Parks Road, Oxford OX1 3AN, UK

## Abstract

A key challenge for volcanological science and hazard management is that few of the world’s volcanoes are effectively monitored. Satellite imagery covers volcanoes globally throughout their eruptive cycles, independent of ground-based monitoring, providing a multidecadal archive suitable for probabilistic analysis linking deformation with eruption. Here we show that, of the 198 volcanoes systematically observed for the past 18 years, 54 deformed, of which 25 also erupted. For assessing eruption potential, this high proportion of deforming volcanoes that also erupted (46%), together with the proportion of non-deforming volcanoes that did not erupt (94%), jointly represent indicators with ‘strong’ evidential worth. Using a larger catalogue of 540 volcanoes observed for 3 years, we demonstrate how this eruption–deformation relationship is influenced by tectonic, petrological and volcanic factors. Satellite technology is rapidly evolving and routine monitoring of the deformation status of all volcanoes from space is anticipated, meaning probabilistic approaches will increasingly inform hazard decisions and strategic development.

## Abstract (web summary)

Deformation is often used in volcano monitoring, but the associated hazard is hard to interpret. Biggs *et al.* analyse 20 years of global satellite data and show a strong statistical link between deformation and eruption, although deformation alone is not necessarily a precursor of imminent eruption.

## Graphical abstract

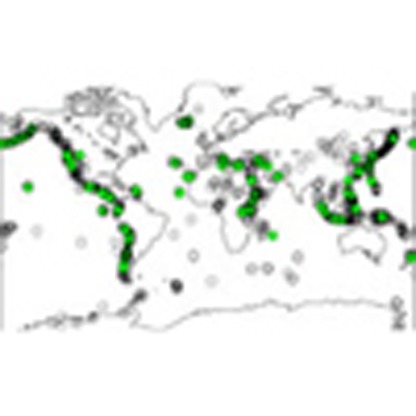

Volcanic unrest is characterized by changes in seismicity, deformation, gas emissions or fumarolic activity^1^. An unrest episode may, or—sometimes crucially—may not lead to eruption^2^. When changes do occur, any lack of baseline information complicates the task of distinguishing between background and precursory activity. Satellite radar (InSAR) can provide high-resolution maps of deformation, allowing the detection of unrest at many volcanoes that may otherwise go unrecognized^3^. At a well-studied volcano, these data form one part of a multiparameter assessment, but are often the only source of information for remote or inaccessible volcanoes^4^.

Although InSAR is burgeoning as a retrospective scientific tool for understanding magmatic plumbing systems^5^, uptake of such data by volcano observatories has been restricted^1^. Critical and timely decisions are often required based on uncertain data and probabilistic tools, such as event trees^6^ and Bayesian Belief Networks^7^, which provide impartial, quantified and defensible information^8^. Only a few frequently erupting and well-monitored volcanoes have a recorded history spanning sufficient periods of unrest and eruption to populate probability tables based on their past behaviour alone.

Rather than relying on this small number of systems, we assess the global statistical relationships using remote observations. Although the multidecadal or shorter timescale of InSAR observations is short compared with many volcano repose periods, by observing a large number of volcanoes at different stages of their eruptive cycle, we adopt an ergodic assumption and substitute geographic coverage for catalogue time length. We then show how tectonic, petrological and volcanic factors influence this relationship.

## Results

### Global contingency table

**Figure 1:**
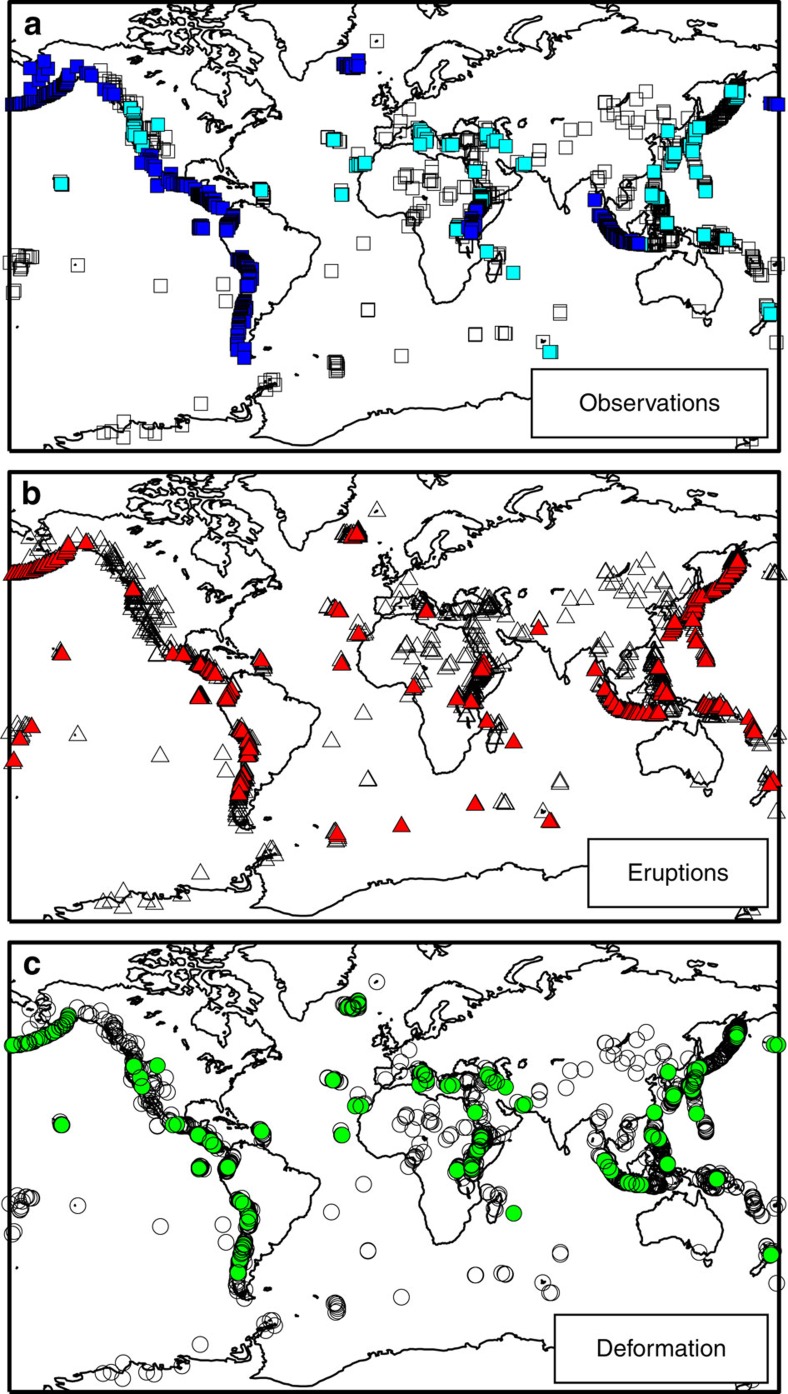
Global distribution of volcanoes. Locations of 1390 named subaerial Holocene volcanoes from the Global Volcano Database^13^ (all symbols) showing (**a**) published satellite studies (filled dark blue: systematic studies including null results; filled pale blue: studies of individual volcanoes, 78% of which report eruption or deformation); (**b**) volcanic eruptions in the Global Volcano Database during the satellite era (filled red), and (**c**) 165 published reports of deformation, including uplift, subsidence, flow compaction, edifice instabilities and all phases of the eruptive cycle (filled green). See Supplementary Tables 1–5.

**Figure 2:**
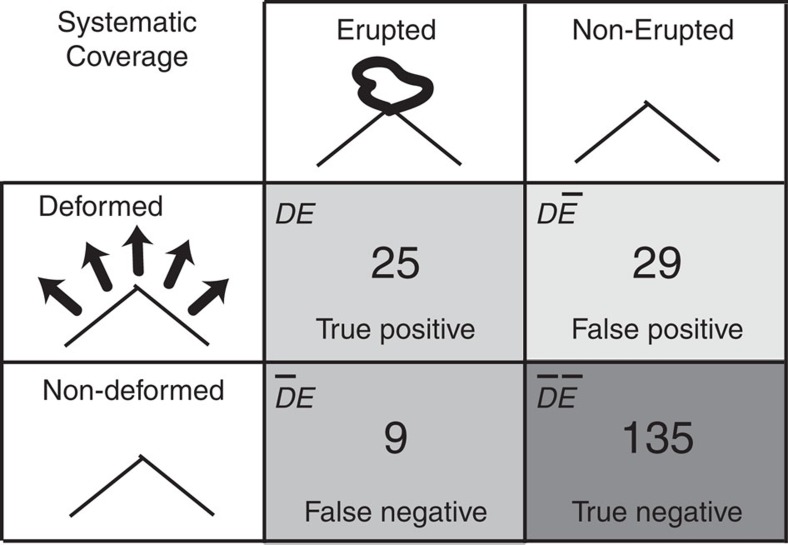
Contingency table linking volcanoes that deformed and erupted. The table reports the number of occurrences in each category out of the 198 systematically observed volcanoes over 18 years (Table 1, Supplementary Table 1). A volcano that ‘deformed’*, D,* is one where at least one period of deformation has been observed with InSAR, while ‘not deformed’, 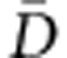, means InSAR measurements were made, but that no deformation was reported. ‘Erupted’, *E,* and ‘not erupted’, 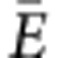, volcanoes are those that erupted or not^13^. See Supplementary Tables 2–5 for details of individual volcanoes.

For each of the 540 volcanoes that comprise systematic InSAR studies (Fig. 1), we define two distinct underlying traits of each volcano: whether they deformed and whether they erupted, and we then use deformed state as a diagnostic test indicator for the probability of at least one eruption having occurred during the period of InSAR observation (Fig. 2). Further details of the catalogue and definitions are provided in the Methods section. To provide global statistics, we select the 198 volcanoes for which there is a full 18-year observation history. Of these, 25 are classified as *DE* (deformed and erupted: in diagnostic test terms, true positives. Supplementary Table 2); 29 are 
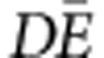
 (deformed but did not erupt; false positives; Supplementary Table 3); 9 are 
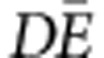
 (erupted, but did not deform; false negatives; Supplementary Table 4) and 135 are 
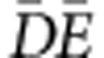
 (did not deform or erupt; true negatives; Supplementary Table 5). Thus, of the 54 volcanoes that deformed, 25 (46%) also erupted during the InSAR observation window, while the proportion of false negatives, 
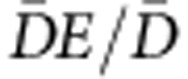
, is very low (∼6%). From a hazard perspective, false negatives are especially concerning due to potential high impacts associated with unanticipated eruptions. Given typically long repose periods^9^ and relatively short observation window, it is not surprising that the most common classification is 
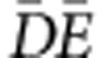
 (68% of volcanoes), but we think this is likely to decrease as observations continue.

From these values, we can quantify the statistical link^10^ between deformation and eruption for the available InSAR data. Following terminology from medical diagnostic testing, we describe this association in terms of positive and negative predictive values (hereafter referred to as *PPV* and *NPV*), but note that ‘predictive’ in this context refers to trait associations and does not imply deformation precedes eruption. The *PPV* of the contingency table (the proportion of volcanoes that deformed between 1992 and 2010 that also erupted; *DE/D*) is 0.46. The *NPV*, that is, the proportion of ‘non-deformed’ volcanoes that did not erupt in the same period (
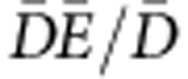
), is 0.94.

Removing cases of deformation attributed to settling and cooling of recent deposits or removing the ∼25% of volcanoes at which measurements are not possible even in systematically studied regions^11^ from the 
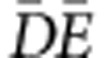
 category does not push *PPV* or *NPV* outside their 95% confidence intervals.

### Volcanological and tectonic influences

The petrological, volcanological and tectonic characteristics of volcanoes vary greatly, and we expect these factors to influence the relationship between deformation and eruption. In the simplest conceptual model routinely applied to geodetic observations, magma supplied to a crustal reservoir from beneath is stored within an elastic medium such that surface deformation rate depends on magma supply rate and storage depth alone^12^. In reality, petrological properties, phase changes, rheological variations, fluid flow, local stress fields and other processes occurring within the complex volcanic environment may also influence or drive deformation. Therefore, numerous factors, some observable but others not, may be expected to correspond with observed deformation patterns to a greater or lesser extent. These include crustal thickness and storage depth; magma composition and volatile content; edifice type and repose period; tectonic setting and plate motion; stress and strain rates; rheology and observations of seismicity and gas release.

**Figure 3:**
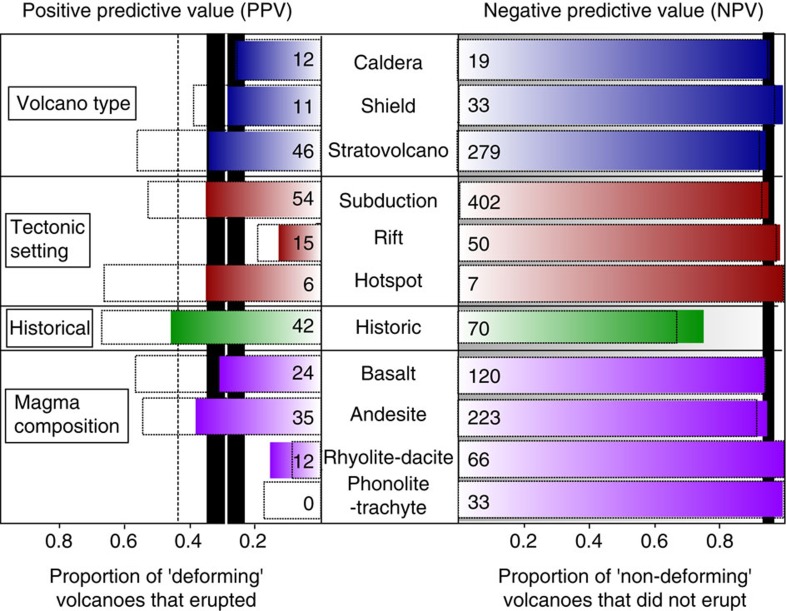
Subdivision according to volcano characteristics. *PPV* and *NPV* subdivided according to (blue) volcano type^13^ (caldera, shield, stratovolcano); (red) tectonic setting (subduction, rift, hotspot); (green) eruptive history^13^; (purple) magma composition^13^ (basalt, andesite, rhyolite-dacite, trachyte-phonolite). Left side: proportion of volcanoes that deformed that also erupted during the observation period (*PPV*); right side: proportion of volcanoes that did not deform that also did not erupt during the observation period (*NPV*). Coloured bars show values for the 3-year period 2007–2010 for all 540 systematically covered volcanoes and the numbers give the sample size for each category; black outlines show the combined 18- year and 3-year data sets. Black vertical bars mark the 95% confidence bounds on the global values (enclosed white bar) on the 3-year data set, vertical dashed line indicates the global values for the combined data sets.

In order to obtain sufficient sampling across all categories, we use an additional 306 tropical volcanoes that were observed for the 3-year period 2007–2010 and select the corresponding 3-year period from the 198 volcanoes that were used for the global compilation. The *PPV* of the 3-year data set of 504 volcanoes is 0.28 and the *NPV* 0.95. Figure 3 then subdivides this catalogue according to volcano type, plate tectonic setting, magma composition and eruptive history^13^ to compare with the 95% confidence intervals of the *PPV* (0.20–0.36) and *NPV* (0.94–0.97) values.

The proportion of eruptions is higher when there has been a historically recorded eruption (0.45), regardless of whether deformation was observed (Fig. 3). Deformation and eruption are most directly linked (a higher *PPV*) for hotspot regions (0.33), basaltic (0.29) and andesitic compositions (0.34). Low volatile content and high magma supply rates contribute to the elastic storage of low compressibility magma in a shallow chamber for a short period before eruption.

The *PPV* is lowest for rhyolite/dacite compositions (0.17), calderas (0.25) and rift settings (0.13). No phonolite or trachyte composition volcanoes deformed during 2007–2010, but the 18-year record shows a low *PPV* for this category too. Magmatic processes associated with the long-term storage of large magma volumes in the shallow crust may produce surface deformation without ensuing eruption (at least within a multidecadal timescale)^14^. Rift settings and low magma flux are associated with longer magma residence times in the shallow crust, more fractionation and crustal assimilation, formation of more evolved magmas and more calderas but less frequent eruptions^9^,^15^,^16^.

We find low NPVs for stratovolcanoes (0.94) and those with basaltic (0.93) and andesitic compositions (0.94) due to a high proportion of 
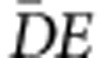
 volcanoes. Eruptions involving little or no juvenile material^17^, highly compressible gas-rich magmas or rapid ascent from great depth may occur with little or no deformation on the weekly to monthly timescales it is possible to detect with InSAR^11^,^14^. Stratovolcanoes are more common on continental crust where the stress field, rigidity contrasts and high parental water contents may contribute to deep storage or compressible magmas, which accommodate pressure changes with little surface deformation^11^,^18^. Processes associated with conduit pressurization and lava dome extrusion also present challenges for InSAR detection^19^.

### Relative timing of deformation and eruption

**Figure 4:**
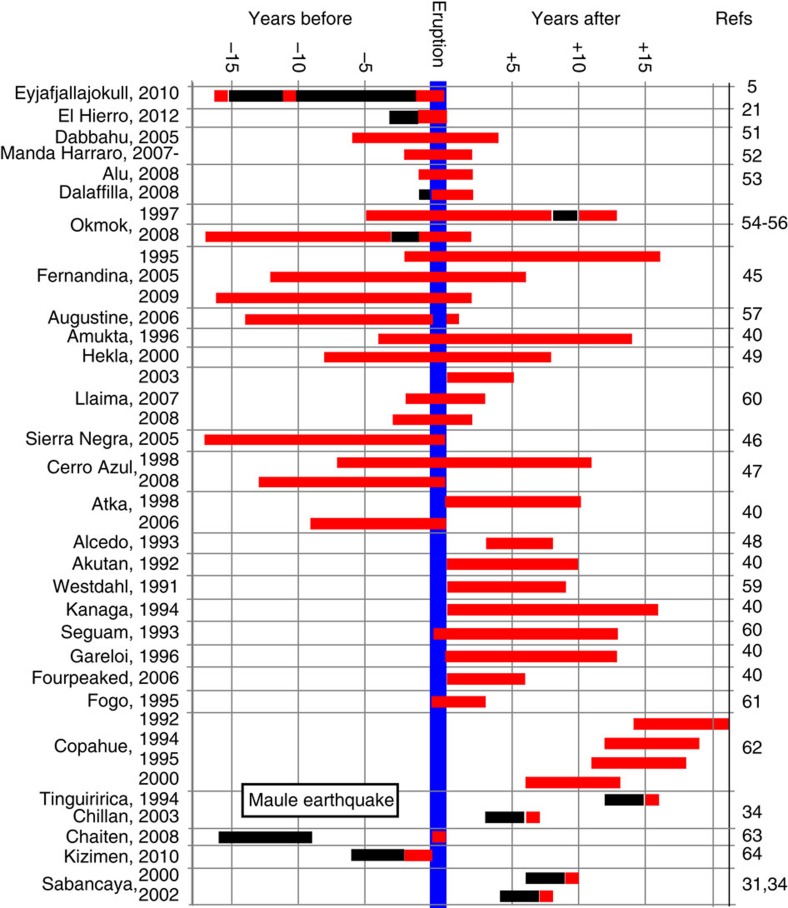
Relative timing of deformation and eruption for volcanoes. For volcanoes where both deformation (measured by InSAR) and eruption are recorded and more than 3 years of observations are reported (See Supplementary Table 7). Volcanoes with persistent lava lakes, continuous effusion or eruptions more than once per 2–3 years are excluded as continuous ground-based data are more appropriate in these cases. Red bar=deformation; black bar=no deformation; no bar=observation not reported. Volcano name and year of the eruption is given to the left of the figure, references are given to the right of the figure. Note: We use the names from the Global Volcano Database^13^ and refer to the entire region containing ‘Dabbahu’, ‘D’Ure’ and ‘Gabho’ as ‘Dabbahu’; the region around ‘Ado Ale’ and ‘Wal’is’ as ‘Manda Hararo’, and ‘Korovin’ and ‘Atka’ as ‘Atka’.

So far, we consider the states ‘deformed’ and ‘erupted’ as two independent, underlying traits, yet defining the temporal and causal links between deformation and eruption is essential for using geodetic observations in a predictive sense. Altering our definitions such that only pre-eruptive deformation is considered a true positive for the 18-year data set (leaving 13 classified as *DE*, and 12 as 
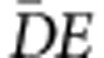
) gives a *PPV* value of 0.31 and a *NPV* of 0.92. Both these values are within the 95% confidence intervals of the *PPV* and *NPV* values for the unfiltered data set. Detailed investigation of the timescale of pre-eruptive deformation based on InSAR observations reveals the strong publication bias towards reporting periods of deformation. Figure 4 covers both individual and systematic studies of *DE* volcanoes and includes all eruptions for which more than 3 years of InSAR data are reported, excluding lava lakes, frequent eruptions (more than one every 3 years) and continuous effusion (Supplementary Table 7). It shows 13 volcanoes that deformed continuously throughout their eruptive cycle and a further 16 where observations began during or after eruption. Deformation is reported to have begun less than a year prior to eruption at Alu in Ethiopia^20^, El Hierro in the Canaries^21^ and Eyjafjallajökull in Iceland^5^. Interestingly, although Alu and Dalafilla erupted simultaneously, Alu showed pre-eruptive deformation but Dalafilla did not^20^. Eyjafjallajökull is unique at present in the InSAR record in that it shows multiple short pulses of uplift, two of which did not end in eruption within a year and one that did^5^,^22^. Time periods during which no deformation occurred (black bars) are rarely reported explicitly. It is likely that observations continued for over 3 years at several of the volcanoes that were excluded from our analysis but only the time periods during which deformation occurred were reported. Infrequent SAR acquisitions may also obscure the temporal relationship between deformation and eruption, especially where deformation is short-lived.

## Discussion

Volcanoes that erupted during the observation window are ∼4 times more likely to have recorded deformation than not (the positive likelihood ratio), meaning this information provides ‘strong’ evidential worth^23^. However, a *PPV* of 0.46 indicates that deformation alone, while worthy of concern, should not be considered a strong diagnostic of imminent eruption. Moreover, the much greater *NPV* is important because although much emphasis is placed on the ability of volcanologists to predict eruptions, often the ability to make a valid negative deduction can be very valuable for hazard and risk decisions.

Statistical tests on ground-based data (primarily seismicity) indicate that pre-eruptive unrest duration varies between volcano types: from 2 days at complex^13^ volcanoes to 5 months at shield volcanoes^24^. Radar images of the majority of the world’s volcanoes are only acquired a few times per year, so deformation on these timescales is only resolvable at the handful of volcanoes for which multiple satellite constellations provide frequent revisits or those with permanent ground-based deformation monitoring. Due to the publication bias towards periods of deformation, we are not yet able to quantify the causal or temporal relationships between the extent, rates, timing, amount and duration of deformation and subsequent eruption using InSAR alone. The forthcoming launches of Sentinel-1 and ALOS-2 will greatly increase temporal and spatial resolution and coverage, enabling more subtle distinctions to be incorporated into pattern analysis.

Nonetheless, even with the current data set it is clear that, although the behaviour of individual plumbing systems is often poorly characterized, the statistics for some subsets of global volcanoes (for example, those on rifts, andesite versus rhyolite and so on, Fig. 3) lie outside the global confidence intervals and are consistent with current understanding of tectonic, petrological and volcanological influences. The short eruption cycles at hotspot volcanoes and those of basaltic and andesitic composition mean that the satellite record typically spans both deformation and eruption resulting in a high *PPV*. For volcanoes with long eruption cycles, the satellite record tends to capture either deformation or eruption but rarely both. In the case of rhyolitic, dacitic, phonolitic and trachytic volcanoes and calderas, the inter-eruption period is characterized by shallow magmatic storage producing regular deformation episodes with infrequent eruptions, resulting in low *PPV*s. In the case of stratovolcanoes, the inter-eruption period is characterized by storage at a range of levels within a thick crust and compressible magmas, producing a high proportion of eruptions without deformation and a relatively low *NPV*. For volcanoes monitored using satellites alone and lacking field measurements, the variation in such diagnostic test statistics associated with globally mapped volcanological and tectonic factors could provide a solid footing for hazard analysis.

The strength of InSAR lies in its global coverage and remote monitoring capabilities; here we quantify the global link between eruption and deformation on multidecadal hazard assessment timescales. Upcoming technological developments promise to improve the frequency of available images and allow us to quantify the causal or temporal relationships between the extent, rates, timing, magnitude and duration of deformation on shorter timescales. Global studies of volcano deformation have potential to be incorporated into strategic hazard assessments^25^, particularly in regions with short historical records^15^.

## Methods

### Volcano catalogue

There are 1390 named subaerial Holocene volcanoes^13^ of which 161 have InSAR-based reports of deformation to date and 620 have reported InSAR observations (Fig. 1). Table 1 and Supplementary Table 1 list systematic studies that include discussion of null results: together they cover >500 volcanoes in the Andes, Central America, Alaska, Africa, Indonesia, Iceland and the Galapagos. The volcanoes in Iceland are not covered by a single systematic study but 85% of them have been included in separate studies of volcanic, seismic, cryospheric or geothermal processes^26^,^27^,^28^,^29^ and those in Galapagos are sufficiently close together to be covered by a single satellite frame. InSAR studies outside these regions tend to focus on individual events, such that the proportion of volcanoes that erupted (32%) or deformed (59%) is significantly higher than the corresponding values of 12% and 17% for the systematic studies. Ground-based geodetic networks provide valuable information at higher temporal resolution and over a longer time period than is accessible using InSAR. However, due to the high logistical and financial overheads, they exist only for a small number of volcanoes, often those that are known to be deforming or erupting. Due to the bias away from ‘null results’ associated with individual studies and ground-based networks, we base our subsequent analysis on systematic InSAR studies alone (Table 1).

Due to the high spatial resolution and coverage, InSAR has been very successful in detecting deformation within volcanically active regions. Sometimes, however, this deformation is up to 25 km from any catalogued volcano summit^13^. In these cases, the deformation has been attributed to the closest listed volcano. Supplementary Table 6 lists deformation attributed to a volcano not in the GVP list of Holocene volcanoes.

### Detection limits

Although InSAR offers an order of magnitude increase in the number of volcanoes that can be observed, several factors limit its ability to detect magmatic processes. The two major limiting factors are as follows: (i) phase delays caused by atmospheric water vapour, particularly the topographically correlated component, and (ii) the loss of signal coherence around snow-capped summits, dense vegetation or steep-sided edifices^30^. Deep sources may cause small magnitude deformation signals that fall below the level of atmospheric noise. For shallow processes, such as the emplacement of viscous plugs, the spatial extent of the signal may be restricted to an incoherent area near the vent^19^. If the system has an air–magma interface or the magma is compressible^18^, volume changes can be accommodated with little or no surface deformation.

Unfortunately, the systematic studies are not uniform in either methodology or error reporting making it difficult to assess how many ‘hidden’ deformation signals exist. Some studies use individual interferograms^31^,^32^, others time series methods^11^,^33^,^34^; some report noise thresholds for each volcano^11^, while others estimate a single value for the survey as a whole^31^,^33^, and some do not mention errors at all^35^. In Central America, time series noise increases by 2 cm for every 1 km of edifice height and measurements are not possible for ∼25% of volcanoes covered^30^. However, there are statistically significant variations in the relationship between the numbers of deformation episodes and historically active volcanoes at volcanic arcs that cannot be attributed to InSAR measurement limitations alone^11^.

### Definitions

Volcanoes show episodic behaviour on timescales ranging between seconds to hundreds of thousands of years^9^ and unrest can take many forms: pulsatory, prolonged, sporadic, reawakening and intra-eruptive^24^. Deformation can occur in the weeks preceding eruption^5^, many years earlier^22^ and for months or years afterwards^36^. Despite this, there is a tendency to conflate the term ‘deformation’ with ‘precursory inflation’, even though many sources of volcano deformation are non-magmatic^36^ and few are directly followed by an eruption.

Although using the satellite archive allows us to consider a large set of volcanoes, the newness of the technology restricts the timescales observed. The current satellite-based deformation record spans 20 years in some temperate regions but only 3 years in the tropics (Table 1) providing a limited snapshot, especially at volcanoes with long eruption cycles. Conversely, rapid cycles of deformation and eruption may not be captured by satellite repeat intervals of 35–46 days^19^,^37^,^38^. Therefore, our initial analysis above avoids subjective judgements regarding the temporal and causal links between deformation and eruption by combining pre-, co- and post-eruptive deformation into a single category, initially discarding for our current purposes the notion of ‘precursory inflation’.

A volcano that ‘deformed’*, D,* is one where at least one period of deformation has been observed with InSAR, while ‘not deformed’, 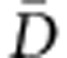, means InSAR measurements were made, but that no deformation was reported. We use the satellite observation windows defined in Table 1 and eruption reports from the Global Volcano Database^13^ to classify each volcano as ‘erupted’, *E,* or ‘not erupted’, 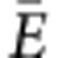 during the observation period. The resulting four classifications shown in Fig. 2, ‘deformed and erupted’ (), ‘deformed but not erupted’ (
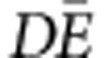
), "not deformed but erupted" (
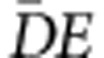
) and ‘not deformed and not erupted’ (
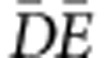
) do not imply any causal link, or even a temporal relationship between any specific eruptions and episodes of deformation (Fig. 2). The subset of observations where deformation precedes eruption is also discussed above.

### Observation window

**Figure 5:**
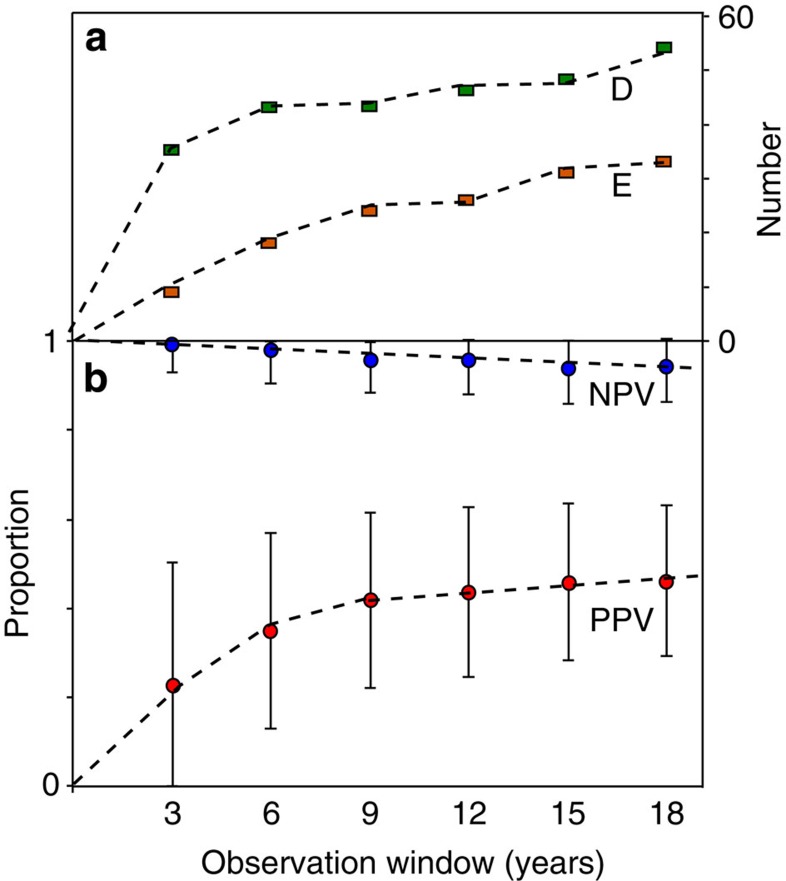
Effect of observation window length. The effect of observation window length is shown on the number of deforming and erupting volcanoes and the values of *PPV* and *NPV*, calculated using subsets of the 198 volcanoes for which there is an 18-year catalogue. We expect the *PPV* to increase to one over geological timescales, but for the timescales over which decisions are required, it stabilizes at values slightly greater than 0.4 for observation windows >9 years. (**a**) Number of volcanoes that erupted (red squares) and number of volcanoes that deformed (green squares). (**b**) Values of *PPV* (red circles) and *NPV* (blue circles) with 95% confidence intervals.

To test the influence of the length of the observation window, we subdivide the observations at the 198 temperate zone volcanoes with an 18-year record into shorter observation windows (3, 6, 9, 12, 15 and 18 years since 1992). The number of volcanoes that deformed and erupted rise as observation window length increases, with a particularly rapid increase over the first 3–6 years (Fig. 5). Over geological timescales, we expect the *PPV* to tend to 1; on shorter timescales, over which hazard decisions are made, the *PPV* is slightly greater than 0.4 for observation windows >9 years (Fig. 5).

## Author contributions

J.B. and S.K.E. carried out the analysis and J.B. wrote the paper with input from all authors. S.K.E., J.B. and T.A.M. conceived the systematic global approach. W.P.A. contributed to the statistical analysis; J.B., S.K.E., Z.L., M.E.P. contributed to the InSAR data set; J.B., R.S.J.S., S.K.E. and T.A.M. contributed to the interpretation.

## Additional information

**How to cite this article:** Biggs, J. *et al.* Global link between deformation and volcanic eruption quantified by satellite imagery. *Nat. Commun.* 5:3471 doi: 10.1038/ncomms4471 (2014).

## Supplementary Material

Supplementary Information Supplementary Tables 1-7 and Supplementary References

## Figures and Tables

**Table 1:** List of regional InSAR studies.

**Region**	**Time period**	**Number**	**References**
East African Rift	1997–2010	38	3,15
Alaska	1992–2010	90	39,40
Central America	2007–2010	117	11,41
Northern Andes	2007–2010	36	32,42
Central Andes	1992–2010	68	31,32,33
Southern Andes	2007–2010	75	31,43
Indonesia	2007–2010	76	33,44
Galapagos	1992–2010	13	45,46,47,48,49
Iceland	1992–2010	27	5,20,22,49,50

These studies report observations at all volcanoes, including those at which no deformation is observed and are compiled to form the catalogue of 540 systematically studied volcanoes used for the statistical analysis of the link between deformation and eruption. Each volcano is assigned two distinct states: either ‘deformed’ or ‘not deformed’ and either ‘erupted’ or ‘not erupted’ which apply to the entire time period regardless of the temporal relationship of deformation and eruption. Further details are given in Supplementary Table 1.
