# Supplementary material for: Global link between deformation and volcanic eruption quantified by satellite imagery
Source: Nat Commun. 2014 Apr 3;5:3471. doi: 10.1038/ncomms4471 (PMC4409635; doi:10.1038/ncomms4471)
Supplement: Supplementary Information — Supplementary Tables 1-7 and Supplementary References [file ncomms4471-s1.pdf]

Supplementary Table 1. Details of individual study regions. The volcano list is taken from the Global Volcanism Programme (GVP) http://www.volcano.si.edu/index.cfm, limited to Holocene volcanoes but with submarine and 'unnamed' volcanoes removed. Each volcano is listed only once, even if there are multiple eruptions or deformation episodes. Accounts of eruptions are taken from the GVP database and references are given for observations of deformation. * Indicates regions used in calculation of 18-year statistics

Region	Time period	Number	E&D	E&ND	D&NE	ND&NE	Reference
East African Rift	1997-2011<br><br>2007-2010	38	1<br><br>1	0<br><br>0	9<br><br>6	28<br><br>31	1,2.
Alaska	1992-2010<br>2007-2010	90*	15<br>4	6<br>2	15<br>20	54<br>64	3, 4.
Central America	2007-2010	117	4	7	1	105	5, 6.
Northern Andes	2007-2010	36	3	2	0	31	7, 8.
Central Andes	1992-2010<br>2007-2010	68*	3<br>1	2<br>0	5<br>7	58<br>60	8,9.<br>10
Southern Andes	2007-2010*	75	2	2	9	62	8, 10.
Indonesia	2007-2010	76	4	7	2	63	11, 12.
Galapagos	1992-2010<br>2007-2010	13*	4<br>2	0<br>0	2<br>4	7<br>7	13, 14, 15
Iceland	1992-2010<br>2007-2010	27*	3<br>1	1<br>1	7<br>7	16<br>18	16. and refs. therein.<br>17,18
18-year	1992-2010	198	25	9	29	135	PPV=0.46<br>NPV=0.94
3-year	2007-2010	540	22	21	56	441	PPV=0.28<br>NPV=0.95

Supplementary Table 2. Volcanoes which both erupted and deformed during observation window. Volcanoes with observed deformation that did not erupt in the observation window. The volcano list is taken from the Global Volcanism Programme (GVP) http://www.volcano.si.edu/index.cfm, limited to Holocene volcanoes but with submarine and 'unnamed' volcanoes removed. Each volcano is listed only once, even if there are multiple eruptions or deformation episodes. Accounts of eruptions are taken from the GVP database and references are given for observations of deformation. The observation window is 2007-2010, except for where regular and useful observations were possible since 1992. Such volcanoes are marked '*' and those in the GVP list are included in the calculation of the 18-year statistics. Volcanoes which were observed to deform only during or after eruption are marked #.

	Volcano	Eruption Date/Style	Deformation Type	Deform 2007-2010?	Ref.
1	Gareloi*#, Alaska	VEI1 in 1996	Flow subsidence	Yes	4
2	Kanaga*#, Alaska	VEI2 in 1994, 1995.	Flow subsidence	Yes	4
3	Cleveland*#, Alaska	Numerous VEI2,3.	Flow subsidence	Yes	3,4
4	Santa María#, C. America	Eruption on SW flank (Santiaguito) since 1922	Flow subsidence	Yes	42
5	Arenal#, C. America	Eruption between 1968-2010(?) VEI 3 at most explosive	Flow subsidence	Yes	43
6	Reventador#, N. Andes	VEI2 in 2001, 2008	Flow subsidence	Yes	23
7	Seguam*#, Alaska	VEI2 in 1992, 1993	Episodic uplift and subsidence	Yes	44
8	Akutan*#, Alaska	VEI2 in 1992	Persistent inflation of 0.5-1.0 cm/yr; Shallow intrusion in 1996.	Yes	3,4
9	Westdahl*#, Alaska	VEI3 in 1991	Persistent uplift at 1-5 cm/yr	Yes	45
10	Fourpeaked*#, Alaska	VEI2 in 2006	Episodic uplift	Yes	4
11	Spurr*#, Alaska	VEI4 in 1992	Episodic uplift	No	4
12	Alcedo*#, Galapagos	VEI1 in 1993	Subsidence of crystallising magma body and landsliding.	Yes	14, 46
13	Sabancaya*#, C. Andes	1990-1991, VEI2 in 2000, VEI3 in 2002.	Deformation 1992-1997 and in 2013, related to earthquakes	No	24
14	Nevados de Chillán*#, C. Andes	VEI 1 in 2003	Only deformation from subsidence triggered by Mw8.8 2010 earthquake	Yes	8,9, 24
15	Bárdarbunga*#, Iceland	Subglacial eruption at Gjalp in 1996.	Dyke injection, then deflation associated with the 1996 Gjalp eruption.	No	25
16	Chaitén#, S.Andes	VEI4 in 2008		Yes	47
17	Sinabung, Indonesia	VEI2 in 2010	Constant uplift at 2.7 cm/yr 2007-2009.	Yes	11
18	Kerinci, Indonesia	VEI in 2007, 2008, 2009 ash/gas plumes < 5 km	Constant uplift at 7.7 cm/yr 2007-2009.	Yes	11

19	Krakatau, Indonesia	VEI 2 in 2007, 2009, 2010	4 cm of uplift for 3 months before eruption; intrusion 4 m-wide dyke after eruption	Yes	48
20	Slamet, Indonesia	VEI2 in 2010; explosive eruptions	Constant uplift at 6.8 cm/yr 2007-2009.	Yes	11
21	Atka*, Alaska	VEI1 in 1995	Episodic inflation	Yes	4
22	Korovin*, Alaska	VEI3 in 1998, VEI1 in 2002,2004,2005,2006	Reports coverage but no deformation	No	3
23	Amukta*, Alaska	VEI1 in 1996, 1997	Subsidence due to contraction of lava flows and/or cooling/degassing of magma	Yes	4
24	Okmok*, Alaska	VEI3 in 1997, VEI4 in 2008	Persistent uplift at variable rate (a few cm/yr to ~20 cm/yr)	Yes	49
25	Makushin*, Alaska	VEI1 in 1993,1995	Episodic uplift and long-term subsidence due to magma intrusion and cooling/degassing	Yes	3
26	Veniaminof*, Alaska	VEI2 in 1993, 2004, 2005; VEI1 in 1995,2002, 2005, 2006, 2008.	Steady uplift at a few cm/yr	Yes	4, 50
27	Augustine*, Alaska	VEI3 in 2005	Slow uplift, pre-eruptive inflation and co-eruptive deflation	Yes	4, 51
28	Colima, Mexico	VEI1 in 1994, VEI3 in 2007-2011	Constant subsidence > 1 cm/year	Yes	21
29	Masaya, C. America	Persistent lava lake, VEI 1 in 2008 ash/ phreatomagmatic	Slow subsidence at ~2 cm/year along ring fault.	Yes	5
30	Fernandina*, Galapagos	VEI2 in 1995, 2005, 2009.	Inflation of radial dyke in 1995; post-eruptive uplift (0.1m) associated with refilling shallow chamber (1998-1999)	Yes	52.
31	Sierra Negra*, Galapagos	VEI3 in 2005	Uplift associated with inflation of shallow sill, 2002-2005.	Yes	15, 53
32	Cerro Azul*, Galapagos	VEI1 in 1998, 2008	pre-eruptive uplift (1992 -1997), post-eruptive subsidence (1998).	Yes	13, 14
33	Láscar*, C.Andes	11 eruptions VEI 1-4 since 1993	Lava dome deformation; Subsidence pyroclastic flow 1993;	Yes	9,5 4,5 5
34	Llaima, S. Andes	VEI2 in 1994, 1995, 1998, 2003, 2007 VEI3 in 2008	Co/post-eruptive in 2008	Yes	56
35	Eyjafjallajökull*, Iceland	VEI4 in 2010	Non-eruptive uplift in 1994, 1999. Uplift	Yes	57

			prior to 2010 eruption.		
36	Hekla*, Iceland	VEI3 in 2000	Long-term inflation (5mm/yr) and co-eruptive deflation of deep source (14-20 km).	Yes	37
37	Ol Doinyo Lengai, E. Africa	Continuous VEI1 VE1 3 in 2007	Intrusion of a 4 km long dyke followed by deflation of a shallow source.	Yes	58
38	Galeras, N. Andes	VEI 1 in 2008	3cm subsidence on northeast flank during 2008 eruption.	Yes	7
39	Tungurahua, N. Andes	VEI3 1999-	20 cm uplift on western flank during 2008 eruption	Yes	59

Supplementary Table 3. Volcanoes with observed deformation that did not erupt in the observation window. The volcano list is taken from the Global Volcanism Programme (GVP) http://www.volcano.si.edu/index.cfm, limited to Holocene volcanoes but with submarine and 'unnamed' volcanoes removed. Each volcano is listed only once, even if there are multiple eruptions or deformation episodes. Accounts of eruptions are taken from the GVP database and references are given for observations of deformation. The observation window is 2007-2010, except for where regular and useful observations were possible since 1992. Such volcanoes are marked '*' and those in the GVP list are included in the calculation of the 18-year statistics.

	Volcano	Deformation	Proposed mechanism	Deforming 2007-2010	Ref.
1	Michoacán-Guan., C. America	Subsidence of recent deposits	Subsidence of recent deposits	Yes	8
2	Lonquimay, S. Andes	Subsidence of recent deposits	Subsidence of recent deposits	Yes	8
3	Novarupta, Alaska	Subsidence of recent deposits	Subsidence of recent deposits	Yes	4
6	Hertali, (aka Haledebi) E. Africa	Variable uplift/subsidence	Shallow magma/hydro	Yes	2
7	Bora-Bericcio, E. Africa	Variable uplift/subsidence	Shallow magma/hydro	Yes	2
8	Alutu, E. Africa	Pulses of uplift + slow subsidence	Shallow magma/hydro	Yes	2
9	Corbetti Caldera, E. Africa	Pulses of uplift (1997-2000; 2010-)	Shallow magma/hydro	Yes	2
10	Silali, E. Africa	Slow subsidence	Shallow magma/hydro	Yes	26
11	Paka, E. Africa	Uplift pulse (2007-8)	Shallow magma/hydro	Yes	1
12	Menengai, E. Africa	Subsidence pulse (1997-2000)	Shallow magma/hydro	No	1
13	Longonot, E. Africa	Uplift pulse (2004-6)	shallow magma/hydro	No	1
14	Suswa, E. Africa	Subsidence pulse (1997-2000)	shallow magma/hydro	No	1
15	Lawu, Indonesia	Variable uplift	shallow magmatic	Yes	11
16	Lamongan, Indonesia	Pulse of uplift 2007	shallow magmatic intrusion	Yes	11
17	Kiska*, Alaska	Episodic subsidence and uplift	hydrothermal	Yes	27
18	Semisopochnoi*, Alaska	Persistent subsidence	cooling of magma/hydro	Yes	4
19	Tanaga*, Alaska	Episodic inflation (none 2007-2010)	magmatic intrusion	No	4
20	Fisher*, Alaska	Persistent subsidence	cooling of magma/hydro	Yes	3
21	Kupreanof*, Alaska	Episodic inflation	magmatic intrusion	Yes	4
22	Aniakchak*, Alaska	Constant subsidence	cooling of magma/hydro	Yes	28
23	Ugashik-Peulik*, Alaska	Uplift pulse 1996-1998	shallow intrusion	No	29
24	Trident*, Alaska	Uplift	shallow magma/hydro	No	30
25	Recheschnoi*, Alaska	Surface uplift (a few cm/yr)		Yes	4
26	Emmons Lake*, Alaska	surface subsidence (1	cooling of magma/hyro	Yes	4

		cm/yr)			
27	Katmai*,<br>Alaska		poor coherence	No	4
28	Yunaska*,<br>Alaska	Subsidence	contraction of surface lava flows	Yes	4
29	Martin*,<br>Alaska	Inflation	magma intrusion	Yes	4
30	Iliamna*,<br>Alaska	Episodic inflation	magma intrusion	No	4
31	Wolf*,<br>Galapagos	Uplift 1992-1997	shallow magma accumulation	Yes	13,14
32	Darwin*,<br>Galapagos	Uplift 1992-1998	shallow magma accumulation	Yes	13,14
33	Near Ticsani*,<br>C. Andes	short subsidence pulse 2005	earthquake swarm induced subsidence	No	31
34	Putana*,<br>C. Andes	short uplift pulse 2009-2010	shallow magma/hydro	Yes	10
35	Cordón de Puntas Negras*<br>C. Andes<br>Aka Cerro Overo	Constant subs 1992-2003; constant uplift 2003-2010	draining and refilling of a distributed shallow crustal reservoir	Yes	10
36	Lastarria*,<br>C. Andes	Variable inflation)~1 cm/yr) (started in ~2001)	hydrothermal	Yes	32,33
37	Robledo*,C.Andes<br>(aka Cerro Blanco)	20 years Persistent Subsidence ~1 cm/yr	hydrothermal	Yes	9, 10
38	Caldera del Atuel,<br>S. Andes	subsidence	triggered by Mw8.8 2010 earthquake (geothermal?	Yes	24
39	Calabozos,<br>S. Andes	subsidence	triggered by Mw8.8 2010 earthquake (geothermal?)	Yes	24
40	Cerro Azul,<br>S. Andes	subsidence	triggered by Mw8.8 2010 earthquake (geothermal?)	Yes	24
41	Laguna del Maule, S. Andes	Variable uplift	Shallow magma/hydro	Yes	8
42	Puyehue-Cordón Caulle, S.Andes	Variable uplift	Combined magma/hydro	Yes	8
43	Cerro Hudson,<br>S. Andes	Uplift at gradually decaying rate.	Refilling shallow magma chamber	Yes	8
44	Tinguiririca.,<br>S. Andes	Subsidence	triggered by Mw8.8 2010 earthquake	Yes	24
45	Copahue, S. Andes	subsidence		Yes	34
46	Krísuvík*,<br>Iceland	Pulses of uplift and subsidence	Combined magma/hydro	Yes	35
47	Hengill*,<br>Iceland	Uplift 1994-1998	Magma intrusion	No	17,36
48	Torfajökull*,<br>Iceland	Constant subsidence	Cooling magma body	Yes	37
49	Kverkfjöll*, Iceland	Uplift 2007-2008	Deep dyke intrusion	Yes	38
50	Askja*,<br>Iceland	Constant subsidence	Cooling magma body.	Yes	39
51	Krafla*,<br>Iceland	Constant subsidence and wide uplift	Shallow cooling, deep refilling	Yes	18,40,41
52	Theistareykjar*, Iceland	Uplift	Magma intrusion	Yes	18,41

Supplementary Table 4. Volcanoes that erupted during the observation window but at which no deformation has been observed. The volcano list is taken from the Global Volcanism Programme (GVP) http://www.volcano.si.edu/index.cfm, limited to Holocene volcanoes but with submarine and 'unnamed' volcanoes removed. Each volcano is listed only once, even if there are multiple eruptions or deformation episodes. Accounts of eruptions are taken from the GVP database and references are given for observations of deformation. The observation window is 2007-2010, except for where regular and useful observations were possible since 1992. Such volcanoes are marked '*' and those in the GVP list are included in the calculation of the 18-year statistics.

	Volcano	Eruption	Data Coverage	Ref.
1	Barren Island, Indonesia	VEI2 in 2008 and 2010.	beyond coverage of systematic survey	11
2	Dempo, Indonesia	VEI1 in 2009	limited data coverage	11
3	Dieng Complex, Indonesia	VEI1 in 2009	limited data coverage	11
4	Merapi, Indonesia	VEI4 in 2010	Reports coverage but no deformation	11
5	Kelut, Indonesia	Dome extrusion 2007-2008,	Reports coverage but no deformation	12
6	Semeru, Indonesia	Continuing eruption since 1967.	Deformation observed with tilt.	19
7	Raung, Indonesia	Ash plumes in 2007-2008,	Reports coverage but no deformation	12
8	Kasatochi*, Alaska	VEI4 in 2008	too small to study	4
9	Shishaldin*, Alaska	VEI3 in 1999	Reports coverage but no deformation	20
10	Pavlof*, Alaska	VEI2 in 1996, 2007	Reports coverage but no deformation	3
11	Chiginagak*, Alaska	VEI1 in 1998	Reports coverage but no deformation	4
12	Wrangell*, Alaska	VEI1 in 1999, 2002	poor coherence	4
13	Popocatépetl, C. America	Continuous cycles of dome forming and collapse.	Reports coverage but no deformation	21
14	Pacaya, C. America	Strombolian activity 1995-6. Lava flows at MacKenney Crater 2004-2010	Reports coverage but no deformation	22
15	San Cristóbal, C. America	VEI 1 in 11/2007, 06/2007, 2008; VEI 2 in 2009. Ash plumes ≤ 9 km.	ALOS 2007-2010, no permanent def. > 2.7 cm/yr.	5
16	Telica, C. America	VEI 1 in 10/2008,07/2008. Phreatic explosions, ash plumes (≤2 km),	ALOS 2007-2010, no permanent def. >1.8 cm/yr.	5
17	Concepción, C. America	VEI 1 in 2007, 2008, VEI 2 in 2007. Explosions, gas/ash plumes (≤1 km)	poor L-band coherence	5
18	Poás, C. America	VEI 1 in 2008, 01/2009, 11/2009. Phreatic explosions, fumerolic activity.	ALOS 2007-2010. no permanent def. >0.9 cm/yr.	5
19	Turrialba, C. America	VEI 2 in 2010 with inc. seismicity/gas	poor L-band coherence	5
20	Nevado del Huila, N. Andes	VEI 2 in 2008; VEI 3 in 2007, 2008-2011.	ALOS 2007-2010. No def. until 2008.	7
21	Guagua Pichincha,	2009 phreatic explosions	2007-2009 ALOS. no deformation	23

	N. Andes			
22	Ubinas*,<br>C. Andes	VEI2 in 2006-2009	Coverage up to 2004, but none spanning eruption	9
23	Irruputuncu,*<br>C. Andes	VEI 2 in 1995	Reports coverage but no deformation	9
24	Planchón-Peteroa,<br>S. Andes	VEI 2 in 2010	Reports coverage but no deformation	24
25	Villarrica,<br>S. Andes	2003-2007.	Reports coverage but no deformation	8
26	Grímsvötn*,<br>Iceland	VEI3 in 1996,1998, 2004,	observations prevented by icecap.	25
27	Redoubt*, Alaska	VEI3 in 2009	GPS observations of deformation, but no InSAR	4

Supplementary Table 5. Volcanoes which are categorized as ND & NE. The volcano list is taken from the Global Volcanism Programme (GVP) http://www.volcano.si.edu/index.cfm, limited to Holocene volcanoes but with submarine and 'unnamed' volcanoes removed. Accounts of eruptions are taken from the GVP database and references are given for observations of deformation.

Alaska	1992-2010	Buldir, Segula, Davidof, Little Sitkin, Takawangha, Bobrof, Moffett, Great Sitkin, Koniuji, Sergief, Chagulak, Herbert, Carlisle, Tana, Uliaga, Kagamil, Vsevidof, Isanotski, Roundtop, Amak, Frosty, Dutton, ,Pavlof Sister, Dana, Stepovak Bay 2, Stepovak Bay 3, Stepovak Bay 4, Black Peak, Yantarni, Kialagvik, Ukinrek Maars, Mageik, Griggs, Snowy Mountain, Denison, Steller, Kukak, Kaguyak, Douglas, Hayes, St. Paul Island, Nunivak Island, Ingakslugwat Hills, St. Michael, Kookooligit Mountains, Imuruk Lake, Buzzard Creek, Sanford, Gordon, Churchill, Edgecumbe, Duncan Canal, Tlevak Strait-Suemez Is., Behm Canal-Rudyerd Bay.
Alaska	2007-2010	As 1992-2010, plus Shishaldin, Chiginagak, Wrangell, Spurr, Korovin, Illiamna, Tanaga, Uguashik-Peulik, Trident, Katmai
C. Andes	1992-2010	Cerro Auquihuato, Sara Sara, Coropuna, Andahua-Orcopampa, Huambo, Nevado Chachani, Cerro Nicholson, El Misti, Huaynaputina, Tutupaca, Yucamane, Nevados Casiri, Tacora, Taapaca, Parinacota, Guallatiri, Tambo Quemado, Isluga, Tata Sabaya, Laguna Jayu Khota, Nuevo Mundo, Pampa Luxsar, Olca-Paruma, Ollagüe, Cerro del Azufre, San Pedro, Sairecabur, Licancabur, Guayaques, Purico Complex, Colachi,<br>Acamarachi, Chiliques, Cerro Overo, Miñiques, Cerro Tujle, Caichinque, Tilocalar, El Negrillar, Pular, La Negrillar, Socompa, Llullaillaco, Cerro Escorial, Cordón del Azufre, Cerro Bayo, Sierra Nevada, Falso Azufre, Nevado de Incahuasi, El Solo, Copiapó, Cerro Tuzgle, Antofagasta de la Sierra, Cerro del Cóndor, Peinado, Tipas, Ojos del Salado
C. Andes	2007-2010	As 1992-2010, plus Sabancaya, 'Near Ticsani', Irruputuncu
Iceland	1992-2010	Snaefellsjökull, Helgrindur, Ljósufjöll, Reykjanes, Brennisteinsfjöll, Hrómundartindur, Grímsnes, Prestahnukur, Hveravellir, Hofsjökull, Tindfjallajökull, Tungnafellsjökull, Fremrinamur, Öraefajökull, Esjufjöll, Jan Mayen.
Iceland	2007-2010	Hengill and Bardabunga
Galapagos	1992-2010	Ecuador, Pinta, Marchena, Genovesa, Santiago, Santa Cruz, San Cristóbal.
E. Africa	1997-2010	Liado Hayk, Dofen, Fentale, Beru, Kone, Boset-Bericha, Bishoftu Volcanic Field, Sodore, Gedamsa,Tullu Moje, East Zway, Butajiri-Silti Field, O'a Caldera, North Island, Central Island, South Island, Marsabit, The Barrier, Namarunu, Segererua Plateau, Emuruangogolak, Korosi, Ol Kokwe, Nyambeni Hills, Homa Mountain, Elmenteita Badlands, Ol Doinyo Eburru, Olkaria
E. Africa	2007-2010	Menengai, Longonot and Suswa
Mexico & C. America	2007-2010	Prieto, Cerro, Pinacate, San Quintín Volcanic Field, Isla San Luis, Jaraguay Volcanic Field, Coronado, Guadalupe, San Borja Volcanic Field, Tres Vírgenes, Isla Tortuga, Comondú-La Purísima, Bárcena, Socorro, Durango Volcanic Field, Isla Isabel, Sangangüey, Ceboruco, Mascota Volcanic Field, Zitácuaro-Valle de Bravo, Jocotitlán, Nevado de Toluca, Chichinautzin, Papayo, Iztaccíhuatl, La Malinche, Serdán-Oriental, Los Humeros, Los Atlixcos, Naolinco Volcanic Field, Cofre de Perote, La Gloria, Las Cumbres, Pico de Orizaba, San Martín, El Chichón, Tacaná, Tajumulco, Almolonga, Atitlán, Tolimán, Acatenango, Fuego, Agua, Cuilapa-Barbarena, Tecuamburro, Jumaytepeque, Moyuta, Flores, Tahual, Cerro Santiago, Suchitán, Chingo, Ixtepeque, Ipala, Chiquimula Volcanic Field, Quezaltepeque, San Diego, Cerro Singüil, Apaneca Range, Santa Ana, Izalco, Coatepeque Caldera, San Salvador, Cerro Cinotepeque, Guazapa, Ilopango, San Vicente, Apastepeque Field, Taburete, Tecapa, Usulután, El Tigre, Chinameca, San Miguel, Laguna Aramuaca, Conchagua, Conchagüita, Isla el Tigre, Isla Zacate Grande, Lake Yojoa, Utila Island, Cosigüina, Rota, Cerro Negro, Las Pilas, Momotombo, Apoyeque, Nejapa-Miraflores, Granada, Mombacho, Zapatera, Maderas, Estelí, Cerro Ciguatepe, Las Lajas, Volcán Azul, Orosí, Rincón de la Vieja, Miravalles, Tenorio, Platanar, Barva, Irazú, Barú, El Valle.

N. Andes	2007-2010	Romeral, Cerro Bravo, Nevado del Ruiz, Santa Isabel, Nevado del Tolima, Machín, Puracé, Sotar, Petacas, Doña Juana, Azufral, Cumbal, Cerro Negro de Mayasquer, Soche, Chachimbiro, Cuicocha, Imbabura, Mojanda, Cayambe, Pululagua, Atacazo, Chacana, Aliso, Sumaco, Illiniza, Cotopaxi, Quilotoa, Chimborazo, Licto, Sangay.
S. Andes	2007-2010	Tupungatito, San José, Maipo, Palomo, Risco Plateado, Infiernillo, Grande Descabezado, San Pedro-Pellado, Nevado de Longaví, Blancas, Lomas, Resago, Payún Matru, Domuyo, Cochiquito Volcanic Group, Tromen, Puesto Cortaderas, Antuco, Trocon,Laguna Mariñaqui, Tolguaca, Tralihue, Laguna Blanca, Sollipulli, Caburgua-Huelemolle, Quetrupillan, Lanín, Huanquihue Group, Mocho Choshuenco, Carrán-Los Venados, Cerro Pantoja, Antillanca Group, Puntiagudo-Cordón Cenizos, Osorno, Tronador, Cayutué-La Viguería, Calbuco, Cuernos del Diablo, Yate, Hornopirén, Apagado, Crater Basalt Volcanic Field, Huequi, Minchinmávida, Yanteles, Corcovado, Palena Volcanic Group, Melimoyu, Puyuhuapi, Mentolat, Cay, Maca, Río Murta, Arenales, Lautaro, Viedma, Aguilera, Reclus, Monte Burney, Palei-Aike Volcanic Field, Fueguino, Aracar.
Indonesia	2007-2010	Seulawah Agam, Peuet Sague, Bur ni Telong, Sibayak,Toba, Imun, Sibualbuali, Lubukraya, Sorikmarapi, Malintang, Sarik-Gajah, Marapi, Tandikat, Talang, Kunyit, Hutapanjang, Sumbing, Pendan, Belirang-Beriti, Bukit Daun, Kaba, Patah, Bukit Lumut Balai, Besar, Ranau, Sekincau Belirang, Suoh, Hulubelu, Rajabasa, Pulosari, Karang, Perbakti-Gagak, Salak, Gede, Patuha, Wayang-Windu, Malabar, Tangkubanparahu, Papandayan, Kendang, Guntur, Tampomas, Galunggung, Talagabodas, Kawah Karaha, Cereme, Sundoro, Sumbing, Ungaran, Telomoyo, Merbabu, Muria, Wilis, Kawi-Butak, Arjuno-Welirang, Penanggungan, Malang Plain, Tengger Caldera, Lurus, Iyang-Argapura, Ijen, Baluran, Talakmau

Supplementary Table 6. Volcanoes with observed deformation that did not erupt in the observation but do not appear in the volcano list and are not included in the statistical analysis. References are given for observations of deformation. The observation window is 2007-2010, except for where regular and useful observations were possible since 1992.

**Volcano**	**Observation**	**Location**	Deform 2007-2010?	Ref.
Uturuncu*, C. Andes	Uplift at 1.1 cm/yr with a moat of subsidence.	Pleistocene volcano, C. Andes	Yes	9,10,60
Lazufre*, C.Andes	Variable inflation (1-3 cm/year) started in 1998	Between Lastarria and Lazufre volcanoes, C. Andes.	Yes	9,32,61
Hualca Hualca*, C.Andes	Inflation from 1992-2002	Stratovolcano not listed in GVP	Yes	9,10
Gelei, E. Africa	Subsidence during a dyke intrusion episode	On the flank of a pre-rift volcanic shield	Yes	62,63.64

Supplementary Table 7: Volcanoes classified as deforming and erupting from both individual and systematic studies, but excluded from Figure 4.

**Persistent lava lakes:**	Erta Ale, Masaya, Nyiragongo
**Continuous effusion:**	Kilauea, Suwanose-jima, Ol Doinyo Lengai,
**Frequent eruptions:**	Soufriere Hills (1997-2010), Tungurahua (1999-), Revantador (2002-), Piton La Fournaise, Karymsky, Cleveland, Veniaminof, Lascar, Etna, Nyamuragira, Miyakejima (2000-),
**<4 years InSAR observations reported:**	Slamet, Usu, Bárdarbunga, Sinabung, Kerinci, Ibu, Kirishima, Unzen, Kuju, Anatahan, Tokachi, Bezymianny, Iwo-jima

Supplementary References.

1. Biggs, J., Anthony, E. Y., & Ebinger, C. J. Multiple inflation and deflation events at Kenyan volcanoes, East African Rift. *Geology*, **37**, 979-982 (2009).
2. Biggs, J., Bastow, I. D., Keir, D., & Lewi, E. Pulses of deformation reveal frequently recurring shallow magmatic activity beneath the Main Ethiopian Rift. *Geochemistry, Geophysics, Geosystems*, **12**, *Q0AB10* (2011).
3. Lu, Z., *et al* Diverse deformation patterns of Aleutian volcanoes from satellite interferometric synthetic aperture radar (InSAR). *Geophysical Monograph Series*, **172**, 249-261 (2007).
4. Lu, Z. & Dzurisin, D. InSAR Imaging of Aleutian Volcanoes: Monitoring a Volcanic Arc from Space. Springer Praxis, (2014).
5. Ebmeier, S. K., Biggs, J., Mather, T. A., & Amelung, F. On the lack of InSAR observations of magmatic deformation at Central American volcanoes. *Journal of Geophysical Research, 118, 2571–2585* (2013).
6. Chaussard, E., Amelung, F., & Aoki, Y. Characterization of open and closed volcanic systems in Indonesia and Mexico using InSAR time series. *Journal of Geophysical Research: Solid Earth*, **118**, 3957-3969 (2013).
7. Parks, M. M., *et al*. Co-eruptive subsidence at Galeras identified during an InSAR survey of Colombian volcanoes (2006–2009). *Journal of Volcanology and Geothermal Research*, **202**, 228-240 (2011).
8. Fournier, T. J., Pritchard, M. E., & Riddick, S. N. Duration, magnitude, and frequency of subaerial volcano deformation events: New results from Latin America using InSAR and a global synthesis. *Geochemistry, Geophysics, Geosystems*, **11**, *Q01003* (2010).
9. Pritchard, M. E., & Simons, M. An InSAR- based survey of volcanic deformation in the central Andes. *Geochemistry, Geophysics, Geosystems*, **5**, *Q02002* (2004).
10. Henderson, S. T., & Pritchard, M. E. Decadal volcanic deformation in the Central Andes Volcanic Zone revealed by InSAR time series. *Geochemistry, Geophysics, Geosystemsm* **14***,*1358–1374 (2013).
11. Chaussard, E., & Amelung, F. Precursory inflation of shallow magma reservoirs at west Sunda volcanoes detected by InSAR. *Geophysical Research Letters*, **39**, *L21311* (2012).
12. Philibosian, B., & Simons, M. A survey of volcanic deformation on Java using ALOS PALSAR interferometric time series. *Geochemistry, Geophysics, Geosystems*, **12** *Q11004* (2011).
13, Amelung, F., Jónsson, S., Zebker, H., & Segall, P. Widespread uplift and 'trapdoor'faulting on Galapagos volcanoes observed with radar interferometry. *Nature*, **407**, 993-996 (2000).
14. Osmanoglu, B., Baker, S., Bagnardi, M., & Amelung, F. The Galápagos Islands seen from space: the contribution of Synthetic Aperture Radar Interferometry (InSAR) to volcano monitoring. *AGU Fall Meeting Abstracts*, **1**, 2268 (2010).
15. Bagnardi, M., Amelung, F., & Poland, M. P. A new model for the growth of basaltic shields based on deformation of Fernandina volcano, Galápagos Islands. *Earth and Planetary Science Letters*, **377**, 358-366 (2013).
16. Sturkell, E. *et al*. Volcano geodesy and magma dynamics in Iceland. *Journal of Volcanology and Geothermal Research*, **150,** 14-34 (2006).
17 Jonsson, S. Anthropogenic and natural deformation on Reykjanes Peninsula, Southwest Iceland, observed using InSAR time-series analysis 1992-2008. *Geophysical Research Abstracts*, **11**, EGU2009-5142-1 (2009).
18. Spaans, K. H., Sigmundsson, F., Hreinsdóttir, S., & Öfeigsson, B. G. High resolution surface deformation measurements in Iceland's Northern Volcanic Zone: Unraveling multiple deformation sources using InSAR and GPS. *Geophysical Research Abstracts*, **14**, EGU2012-10604 (2012).
19. Iguchi, M., Yakiwara, H., Tameguri, T., Hendrasto, M. & Hirabayashi, J.-I. Mechanism of explosive eruption revealed by geophysical observations at the Sakurajima, Suwanosejima and Semeru volcanoes, *Journal of Volcanology Geothermal Research,* **178**, 1-9 (2008).
20. Moran, S. C., Kwoun, O., Masterlark, T., & Lu, Z. On the absence of InSAR-detected volcano deformation spanning the 1995–1996 and 1999 eruptions of Shishaldin Volcano, Alaska. *Journal of Volcanology and Geothermal Research*, **150**, 119-131 (2006).

21. Pinel, V., *et al*. The challenging retrieval of the displacement field from InSAR data for andesitic stratovolcanoes: Case study of Popocatepetl and Colima volcano, Mexico. *Journal of Volcanology and Geothermal Research*, **200**, 49-61 (2011).
22. Zebker, H. A., Amelung, F. & Jonsson, S. Remote Sensing of Volcano Surface and Internal Processes Using Radar Interferometry, in *Remote Sensing of Active Volcanism* (eds P. J. Mouginis-Mark, J. A. Crisp and J. H. Fink), American Geophysical Union, Washington, D. C. (2013).
23. Mothes, P., *et al.* Survey of Volcanic Activity in Ecuador using L-band SAR. In *AGU Fall Meeting Abstracts*, **1**, 2055 (2008).
24. Pritchard, M. E., Jay, J.A., Aron, F., Henderson, S.T. & Lara, L. E. Subsidence at southern Andes volcanoes induced by the 2010 Maule, Chile earthquake, *Nature Geoscience*, **6**, 632–636 (2013).
25. Pagli, C. *et al*. Crustal deformation associated with the 1996 Gjálp subglacial eruption, Iceland: InSAR studies in affected areas adjacent to the Vatnajökull ice cap. *Earth and Planetary Science Letters*, **259**, 24-33 (2007).
26. Biggs, J., Robertson, E., & Mace, M. ISMER—Active Magmatic Processes in the East African Rift: A Satellite Radar Perspective. In *Remote Sensing Advances for Earth System Science,* pp. 81-91, Springer Berlin Heidelberg (2013).
27. Lu, Z., Masterlark, T., Power, J., Dzurisin, D., & Wicks, C. Subsidence at Kiska volcano, western Aleutians, detected by satellite radar interferometry. *Geophysical Research Letters*, **29**, 2-1 (2002).
28. Kwoun, O. I., Lu, Z., Neal, C., & Wicks, C. Quiescent deformation of the Aniakchak Caldera, Alaska, mapped by InSAR. *Geology*, **34**, 5-8 (2006).
29. Lu, Z., *et al* Magmatic inflation at a dormant stratovolcano: 1996–1998 activity at Mount Peulik volcano, Alaska, revealed by satellite radar interferometry. *Journal of Geophysical Research,* **107**, ETG 4-1– ETG 4-13 (2002).
30. Lu, Z., *et al*. Deformation of New Trident Volcano measured by ERS- 1 SAR interferometry, Katmai National Park, Alaska. *Geophysical Research Letters*, **24**, 695-698 (1997).
31. Holtkamp, S. G., Pritchard, M. E., & Lohman, R. B. Earthquake swarms in South America. *Geophysical Journal International*, **187**, 128-146 (2011).
32. Ruch, J. *et al.* Stress transfer in the Lazufre volcanic area, central Andes. *Geophysical Research Letters*, **36**, *L22303* (2009).
33. Froger, J. L., Rémy, D., Bonvalot, S., & Legrand, D. Two scales of inflation at Lastarria-Cordon del Azufre volcanic complex, central Andes, revealed from ASAR-ENVISAT interferometric data. *Earth and Planetary Science Letters*, **255**, 148-163 (2007)
34. Velez, M. L., Euillades, P., Caselli, A., Blanco, M., & Díaz, J. M. Deformation of Copahue volcano: Inversion of InSAR data using a genetic algorithm. *Journal of Volcanology and Geothermal Research*, **202**, 117-126 (2011).
35. Michalczewska, K., *et al.* The inflation and deflation episodes in the Krísuvík geothermal area. *AGU Fall Meeting Abstracts* **1**, 2589 (2011).
36. Clifton, A. E., Sigmundsson, F., Feigl, K. L., Guðmundsson, G., & Árnadóttir, T. Surface effects of faulting and deformation resulting from magma accumulation at the Hengill triple junction, SW Iceland, 1994–1998. *Journal of Volcanology and Geothermal Research*, **115**, 233-255 (2002).
37. Ofeigsson, B. G., Hooper, A., Sigmundsson, F., Sturkell, E., & Grapenthin, R. Deep magma storage at Hekla volcano, Iceland, revealed by InSAR time series analysis. *Journal of Geophysical Research.***116,** B05401 (2011).
38. Hooper, A. *et al* Increased capture of magma in the crust promoted by ice-cap retreat in Iceland. *Nature Geoscience*, **4**, 783-786 (2011).
39. De Zeeuw-van Dalfsen, E., Pedersen, R., Hooper, A., & Sigmundsson, F. Subsidence of Askja caldera 2000–2009: modelling of deformation processes at an extensional plate boundary, constrained by time series InSAR analysis. *Journal of Volcanology and Geothermal Research*, **213,** 72-82 (2012).
40. De Zeeuw- van Dalfsen, E., Pedersen, R., Sigmundsson, F., & Pagli, C. Satellite radar interferometry 1993–1999 suggests deep accumulation of magma near the crust- mantle boundary at the Krafla volcanic system, Iceland. *Geophysical Research Letters*, **31**, *L13611* (2004).

41. Metzger, S., Jónsson, S., & Geirsson, H. Locking depth and slip- rate of the Húsavík Flatey fault, North Iceland, derived from continuous GPS data 2006–2010. *Geophysical Journal International*, **187**, 564-576 (2011).
42. Ebmeier, S. K., *et al.*, Measuring large topographic change with InSAR: Lava thicknesses, extrusion rate and subsidence rate at Santiaguito volcano, Guatemala. *Earth and Planetary Science Letters*, **335**, 216-225 (2012).
43. Ebmeier, S. K., Biggs, J., Mather, T. A., Wadge, G., & Amelung, F. Steady downslope movement on the western flank of Arenal volcano, Costa Rica. *Geochemistry, Geophysics, Geosystems*, **11**, *Q12004* (2010).
44. Lee, C-W. Lu, Z., Won, J-S., Jung, H-S, Dzurisin, D., Dynamic deformation of Seguam Island, Alaska, 1992–2008, from multi-interferogram InSAR processing, *Journal of Volcanology and Geothermal Research*, **260**, 43–51 (2013).
45. Lu, Z., *et al.* Aseismic inflation of Westdahl volcano, Alaska, revealed by satellite radar interferometry. *Geophysical Research Letters*, **27**, 1567-1570 (2000).
46. Hooper, A.*,* Segall*,* P. *&* Zebker, H.*,* Persistent scatterer interferometric synthetic aperture radar for crustal deformation analysis, with application to Volcán Alcedo, Galápagos*, Journal of Geophysical Research,* **112***,* B07407 *(*2007).
47. Wicks, C., de La Llera, J. C., Lara, L. E., & Lowenstern, J. The role of dyking and fault control in the rapid onset of eruption at Chaitén volcano, Chile. *Nature*, **478**, 374-377 (2011).
48. Agustan, A., Kimata, F., Pamitro, Y. E., & Abidin, H. Z. Ground deformation of Krakatau Volcano, Indonesia, detected by InSAR: June 2007 to February 2009. *AGU Fall Meeting Abstracts*, **1**, 692 (2009).
49. Lu, Z., Dzurisin, D., Biggs, J., Wicks, C., & McNutt, S. Ground surface deformation patterns, magma supply, and magma storage at Okmok volcano, Alaska, from InSAR analysis: 1. Intereruption deformation, 1997–2008. *Journal of Geophysical Research,***115**, (2010).
50. Fournier, T.J., and Freymueller, J.T., Inflation detected at Mount Veniaminof, Alaska, with campaign GPS: *Geophysical Research Letters*, **35**, L20306 (2008).
51. Cervelli, P. *et al,* The geodetic perspective on the 2006 eruption of Augustine Volcano, U.S. Geological Survey Professional Paper ***1769***, 427–452 (2010).
52. Chadwick Jr, W. W., *et al,* The May 2005 eruption of Fernandina volcano, Galápagos: The first circumferential dike intrusion observed by GPS and InSAR. *Bulletin of volcanology*, **73**, 679-697 (2011).
53. Chadwick, W. W. *et al.* A volcano bursting at the seams: inflation, faulting, and eruption at Sierra Negra Volcano, Galápagos. *Geology*, **34**, 1025-1028 (2006).
54. Pavez, A. *et al*. Insight into ground deformations at Lascar volcano (Chile) from SAR interferometry, photogrammetry and GPS data: Implications on volcano dynamics and future space monitoring. *Remote Sensing of Environment*, **100**, 307-320 (2006).
55. Whelley, P. L. *et al.* Post-depositional fracturing and subsidence of pumice flow deposits: Lascar volcano, Chile, Bulletin of Volcanology, **74**, 511-531 (2011).
56. Bathke, H., Shirzaei, M., & Walter, T. R. Inflation and deflation at the steep- sided Llaima stratovolcano (Chile) detected by using InSAR. *Geophysical Research Letters*, **38** *L10304 (2011)*.
57. Sigmundsson, F. *et al.*, Intrusion triggering of the 2010 Eyjafjallajokull explosive eruption. *Nature*, **468**, 426-430 (2010).
58. Biggs, J., Chivers, M. & Hutchinson, M, Surface deformation and stress interactions during the 2007-2010 sequence of earthquake, dyke intrusion and eruption in Northern Tanzania. Geophysical Journal International, **195**, 16-26 (2013).
59. Biggs, J. *et al.* Stratovolcano growth by co- eruptive intrusion: the 2008 eruption of Tungurahua, Ecuador. *Geophysical Research Letters*, **37**, *L21302* (2010).
60. Fialko, Y., & Pearse, J. Sombrero uplift above the Altiplano-Puna magma body: evidence of a ballooning mid-crustal diapir. *Science*, **338**, 250-252 (2012)
61. Ruch, J., & Walter, T. R. Relationship between the InSAR-measured uplift, the structural framework, and the present-day stress field at Lazufre volcanic area, central Andes. *Tectonophysics*, **492**, 133-140 (2010).
62. Baer, G., Hamiel, Y., Shamir, G., & Nof, R. Evolution of a magma-driven earthquake swarm and triggering of the nearby Oldoinyo Lengai eruption, as resolved by InSAR, ground observations

and elastic odelling, East African Rift, 2007. *Earth and Planetary Science Letters*, **272**, 339-352 (2008).
63. Calais, E. *et al*, Strain accommodation by slow slip and dyking in a youthful continental rift, East Africa. *Nature*, **456**, 783-787 (2008).
64. Biggs, J., Amelung, F., Gourmelen, N., Dixon, T. H., & Kim, S. W. InSAR observations of 2007 Tanzania rifting episode reveal mixed fault and dyke extension in an immature continental rift. *Geophysical Journal International*, **179**, 549-558 (2009).
